# Renal doppler ultrasound: comparison of measurements sampled in different anatomical locations

**DOI:** 10.1007/s10877-026-01413-3

**Published:** 2026-02-20

**Authors:** Rasmus Aagaard, Johan Lyngklip Hermansen, Simone Krogh Christensen, Peter Juhl-Olsen

**Affiliations:** 1 Department of Cardiothoracic and Vascular Surgery, Anaesthesia Section, Aarhus University Hospital, Aarhus, Denmark; 2 Department of Anesthesiology and Intensive Care, Viborg Regional Hospital, Viborg, Denmark; 3 Department of Emergency Medicine, Aarhus University Hospital, Aarhus, Denmark

**Keywords:** Doppler ultrasound, Resistive index, Renal venous stasis index, Renal venous flow pattern, Acute kidney injury.

## Abstract

Renal Doppler ultrasound can be used to predict acute kidney injury and assess venous congestion. There is no consensus on which renal vessels should be used for Doppler sampling. This study compares renal Doppler ultrasound measurements obtained in renal hilar and interlobar arteries and veins in patients recovering from open-heart surgery.This secondary analysis included data from 99 patients who underwent cardiac surgery. Doppler ultrasound was performed on both hilar and interlobar vessels in each kidney and renal venous flow pattern, renal venous stasis index (RVSI), and renal arterial resistive index (RI) were assessed.For renal venous flow pattern Kappa was 84% for hilar vs. interlobar measurements, 63% for hilar measurements on either side, and 77% for interlobar measurements on either side. When comparing RVSI the biases ranged from 0.2% to 4.8%, limits of agreement were consistently wide, and percentage error ranged from 96% to 133%. The comparisons of RI showed biases from 0.1% to 0.4% with limits of agreement consistently narrow. Percentage error ranged from 9.9% to 12.3%In a population of postoperative open-heart cardiac surgery patients, comparable results can be expected when sampling renal venous flow pattern from hilar and interlobar veins. Overall low bias but also low precision can be expected when sampling RVSI in hilar and interlobar veins on either side. In regard to RI, low bias and high precision can be expected when sampling from hilar and interlobar arteries on either side.

**Supplementary Information:**

The online version contains supplementary material available at 10.1007/s10877-026-01413-3.

## Introduction

Renal Doppler ultrasound is a non-invasive tool that has gained increasing clinical relevance for predicting acute kidney injury and for assessing venous congestion. Doppler ultrasound measurements of the renal artery resistive index predict acute kidney injury after cardiac surgery [1, 2]. Renal venous flow patterns, particularly when assessed together with hepatic- and portal venous flow using the Venous Excess Ultrasound (VExUS) grading system, may indicate venous congestion and also serve as a predictive marker for acute kidney injury [2–4].

Venous flow patterns are to some extent reflections of how right atrial pressure fluctuates during each cardiac cycle. Specifically, there is a pressure peak during atrial contraction, termed the “a wave”, and another pressure peak at the end of ventricular filling, termed the “v wave”. Additionally, there are pressure decreases during initial atrial filling corresponding to ventricular contraction, and during right ventricular filling, termed the “x-descent” and “y-descent”, respectively. These fluctuations in pressure are directly reflected in corresponding changes in hepatic venous flow, while the pressure effect further up-stream in the renal veins may be attenuated due to high venous compliance. Thus, in healthy individuals the renal venous flow is usually continuous with only slight pulsatile fluctuations. However, as the filling pressure in the veins and right heart increases, venous compliance is reduced and fluctuations in right atrial pressure are better transmitted to the renal veins, resulting in periotic flow interruptions. Moderately elevated filling pressure is associated with interruptions in renal venous flow during both the “a-wave” and “v-wave” and this flow pattern can be described as biphasic. In the case of severely elevated filling pressure, forward renal venous flow is only present during the y-descent and this flow pattern is monophasic. It is therefore evident, that the resulting venous flow profiles represent a complex result of the right atrial pressure curve morphology, venous compliance and the renal venous capillary outflow pressure [5–7].

The changes in renal venous flow with increasing venous filling pressures can also be described using the renal venous stasis index, which is the proportion of the cardiac cycle during which there is no renal venous flow [8].

Renal arterial flow can be evaluated with the resistive index. The resistive index reflects a multitude of flow- and vessel characteristics, including the resistance to arterial flow [9]. The resistive index is a mathematical expression of the ratio between the difference in peak systolic flow and the end-diastolic flow, and the peak systolic flow. Normal values for resistive index are between 0.5 and 0.7 whereas higher values indicate greater resistance [9]. 

The renal venous and renal arterial vascular anatomies differ slightly. The renal arteries branch into segmental arteries immediately before entering the renal hilum. In the renal sinus, the segmental arteries give rise to the interlobar arteries when following the renal lobes of the renal medulla. As the interlobar arteries enter the tissue proper between the renal medulla and cortex, the interlobar arteries form the arcuate arteries, which run in an arch-like manner in this space. The arcuate arteries give rise to the interlobular arteries that branch into afferent arterioles. The renal veins follow the arcuate and interlobar arteries but have no clear segmental organization. In contrast to the arterial organization, there are widespread venous anastomoses throughout the kidney [5, 10].

There is a lack of consensus regarding the choice of anatomical Doppler sample location. Sampling from hilar [2], interlobar [11], and arcuate [1] vessels have been used, while other studies sample from the cortico-medullary junction [4]. Also, some studies have used only Doppler measurements from the right kidney [8], while others have prioritized measurements from the vessels most clearly visualized [2].

Limited published data describes how the choice of anatomical Doppler sample location impacts Doppler ultrasound recordings. Data based on results from young, healthy volunteers indicate that little difference in the resistive index can be expected when sampling from arteries near the renal hilum as opposed to interlobar arteries [12]. Similar results were found when comparing resistive indices from the right and left kidney [13]. Data on the effects of sampling location for Doppler ultrasound renal venous flow patterns is completely lacking.

The primary objective of this study was to compare arterial- and venous renal Doppler ultrasound measurements obtained in hilar renal vessels with renal Doppler ultrasound measurements from interlobar renal vessels in adult patients recovering from open heart surgery. A secondary objective was to compare arterial- and venous renal Doppler ultrasound measurements performed on the right- and left kidney.

We hypothesized that the biases between continuous renal Doppler ultrasound measurements are lower than 10% with a percentage error less than 30%, and, for categorical measurements, that the concordance rates are above 90% when comparing measurements obtained from the

- Hilar and interlobar vessels in the kidney with the best image quality.
- Hilar vessels in the right- and left kidney.
- Interlobar vessels in the right- and left kidney.

## Methods

This was a secondary analysis of prospectively collected data obtained for a previously published clinical study. The study was approved by the Regional Ethical Committee, and signed consent was obtained from all participants. Patients above 18 years of age with one or more known risk ­factors for postoperative acute kidney injury undergoing elective open-heart, on-pump cardiac surgery at Aarhus University Hospital, Denmark were eligible for inclusion. Risk factors for acute kidney injury included age > 70 years, New York Heart Association classification 3 or 4, insulin dependent diabetes mellitus, preoperative estimated glomerular filtration rate < 60 ml/min/1.72m^2^, left ventricular ejection fraction < 35%, diagnosed peripheral vascular disease, re-do surgery, valve surgery except isolated aortic valve, pulmonary thromboendarectomy. Please see Hermansen et al. for further details [2].

### Renal doppler ultrasound

Renal Doppler ultrasound examinations were performed on the first postoperative day after open-heart cardiac surgery by one of two sonographers with more than 5 years of experience in Doppler ultrasound. Prior to study enrollment, patients were informed that the ultrasound examinations were extensive and that they could refuse further scans at any time. Also, the ultrasound examinations could be prematurely interrupted if patient care or other time essential interventions interfered. A Vivid S6 or S70N ultrasound system (GE Healthcare, Horten, Norway) with a 4 C- or C1–6 curvilinear probe (GE Healthcare) was used. The right kidney was examined first, and the left kidney subsequently. Electrocardiograms for use in later analyses were recorded. Patients were placed in a supine position and scanned using a lateral approach. Images were analyzed in Echopac (Version 202, GE Healthcare, Horten, Norway) by a single observer blinded to the results of previous within patient analyses. Flow values were averaged over three cardiac cycles. In case of atrial fibrillation, flow values were averaged over three index beats (the beat following 2 preceding cardiac cycles of equal duration). When comparing Doppler ultrasound indices obtained from the hilar and interlobar vessels, measurements obtained from the kidney providing the best image quality were used for each patient. For each Doppler ultrasound measurement, the sonographer evaluated if image quality was sufficient for reliable measurements. If image quality was deemed insufficient, no Doppler ultrasound data was recorded.

### Doppler ultrasound indices

*Venous flow pattern* was categorized as either, 1) continuous, meaning continuous flow throughout the cardiac cycle only allowing a short end-diastolic pause immediately after the p-wave in the electrocardiogram (caused by the “a-wave” due to atrial contraction); 2) biphasic, meaning flow present with two pauses in one cardiac cycle; or 3) monophasic, meaning flow present only in ventricular diastole.

*Renal venous stasis index* is the proportion of the cardiac cycle during which there is no renal venous flow and was calculated as follows:

1 $$\begin{gathered} \:{\mathrm{Renal}}\:{\mathrm{venous}}\:{\mathrm{stasis}}\:{\mathrm{index}}\:\hfill \\ {\text{ = }}\frac{{{\mathrm{cardiac}}\:{\mathrm{cycle}}\:{\mathrm{time}}\:{\text{ - }}\:{\mathrm{venous}}\:{\mathrm{flow}}\:{\mathrm{time}}}}{{{\mathrm{index}}\:{\mathrm{cardiac}}\:{\mathrm{cycle}}\:{\mathrm{time}}}} \hfill \\ \end{gathered}$$

*Resistive index* is a measure of the fractional difference in arterial flow velocities and was calculated as follows:

2 $$\begin{gathered} \:{\mathrm{Resistive}}\:{\mathrm{index}}\: \hfill \\ {\text{ = }}\:\:\frac{{{\mathrm{peak}}\:{\mathrm{systolic}}\:{\mathrm{velocity}}\:{\text{ - }}\:{\mathrm{end}}\:{\mathrm{diatolic}}\:{\mathrm{velocity}}}}{{{\mathrm{peak}}\:{\mathrm{systolic}}\:{\mathrm{velocity}}}} \hfill \\ \end{gathered}$$

### Statistical analysis

As this secondary analysis of a trial represents a convenience sample, no formal sample size calculation was performed. Measurements of renal arterial resistive index and renal stasis index obtained in different anatomical locations were compared using Bland-Altman analyses and calculation of percentage error. For Band-Altman analyses, the difference of each set of measurements was plotted against the mean with the corresponding 95% confidence interval (CI) along with 95% limits of agreement (LoA). The distribution of the differences in each set of measurements was assessed for normality by visual inspection of histograms and QQ-plots. Percentage error was calculated as $\:\frac{1.96\cdot\:SD\:of\:bias}{mean\:of\:all\:measurements\:}\cdot\:100$.

The renal venous flow pattern is a categorical variable, and comparisons were analyzed by calculating Cohen’s Kappa and concordance rates. The Cohen’s Kappa values were interpreted as suggested by McHugh. Kappa values from 40% to 59% suggested weak agreement, 60% to 79% suggested moderate agreement, values above 80% suggested strong agreement, and values above 90% suggested almost perfect agreement [14]. 

Analyses were performed using Stata 18.0 (StataCorp. 2023. Stata Statistical Software: Release 18. College Station, TX: StataCorp LLC.)

## Results

The original study included 100 patients and ultrasound data was obtained from 99 patients due to sonographer unavailability for one patient. See Table 1 for patient characteristics. In 21 patients, imaging was not attempted on the left kidney because the exam was stopped on patient request or because other interventions related to patient care had to be prioritized. Table 2 shows the number of patients with image quality sufficient for reliable arterial and venous flow measurements. The results of all Doppler ultrasound measurements in each anatomical sample location are depicted in Table 3.

**Table 1:** Patient characteristics

-	All patients (*n* = 99)
Age, years	72 (64; 74)
Male, n	79 (80%)
BMI (kg/m2)	27.9 (24.9; 30.6)
EuroSCORE II	1.67 (1.19; 2.56)
Hypertension, n	71 (72%)
Diabetes mellitus, n	22 (22%)
Active smoking, n	15 (15%)
eGFR < 60 ml/min/1.73 m2, n	27 (27%)
LVEF < 50%, n	33 (33%)
Pulmonary hypertension, n	17 (17%)
*Surgery type*	
CABG, n	46 (46%)
Aortic valve, n	10 (10%)
Mitral valve, n	12 (12%)
Combination (Two procedures), n	24 (24%)
Other, n	8 (8%)
*Hemodynamic characteristics*	
Fluid balance (L)*	1.1 (0.5; 1.9)
Mean arterial pressure (mmHg)	79.5 (72.8; 84.8)
Heart rate (beats∙min-1)	80 (69; 83)
Central venous pressure (mmHg)	11 (8; 13)
Mechanical ventilation, n	17 (17%)
Norepinephrine, n	28 (28%)
Dobutamine, n	9 (9%)

Data are given as median interquartile range (IQR) or number (n) and percentage. CABG (coronary artery bypass grafting), BMI (body mass index), LVEF (left ventricular ejection fraction), eGFR (estimated glomerular infiltration rate), EuroSCORE II (European system for Cardiac Operative Risk Evaluation II)*Fluid balance refers to the period from start of surgery to the time of the ultrasound examination. It is the calculated balance between fluid in- and output for each patient, including urine output, bleeding, estimated loss through perspiration, and all intravenous and oral inputs. Perspiration is estimated at 10 ml/kg/h during the first hour of surgery and 5 ml/kg/h during subsequent hours of surgery. Postoperatively, it is estimated at 0,41 ml/kg/h

**Table 2:** Number of scans with image quality sufficient for reliable arterial and venous flow measurements

-	Number of patients	% of attempted
*Right kidney – attempted in 99 patients*		
Reliable arterial Doppler measurements obtained		
Hilar renal arteries	89	90%
Interlobar renal arteries	95	96%
Reliable venous Doppler measurements obtained		
Hilar renal veins	90	91%
Interlobar renal veins	96	97%
*Left kidney – attempted in 78 patients*		
Reliable arterial Doppler measurements obtained		
Hilar renal arteries	47	60%
Interlobar renal arteries	61	78%
Reliable venous Doppler measurements obtained		
Hilar renal veins	42	54%
Interlobar renal veins	56	72%

**Table 3:** Renal doppler ultrasound measurements

-	Resistive index, Mean (95% CI)	Venous flow pattern	N (%)	Renal venous stasis index, Median [IQR]
Right kidney, hilar vessel	0.73 (0.72–0.74) (89 Measurements)	Continuous	41 (41)	0.22 [0–0.36] (90 Measurements)
		Biphasic	44 (44)	
		Monophasic	5 (5)	
		Not obtained	9 (9)	
Left kidney, hilar vessel	0.74 (0.73–0.76) (47 Measurements)	Continuous	21 (21)	0.17 [0–0.38] (42 Measurements)
		Biphasic	16 (16)	
		Monophasic	5 (5)	
		Not obtained	57 (58)	
Right kidney, interlobar vessel	0.73 (0.72–0.74) (95 Measurements)	Continuous	49 (49)	0.16 [0–0.34] (96 Measurements)
		Biphasic	42 (42)	
		Monophasic	5 (5)	
		Not obtained	3 (3)	
Left kidney, interlobar vessel	0.74 (0.72–0.75) (61 Measurements)	Continuous	32 (32)	0.17 [0–0.38] (56 Measurements)
		Biphasic	19 (19)	
		Monophasic	5 (5)	
		Not obtained	43 (43)	

Measurement values obtained in different anatomical locations. CI - Confidence interval; IQR - Interquartile range

### Renal venous flow pattern

**Fig. 1:**
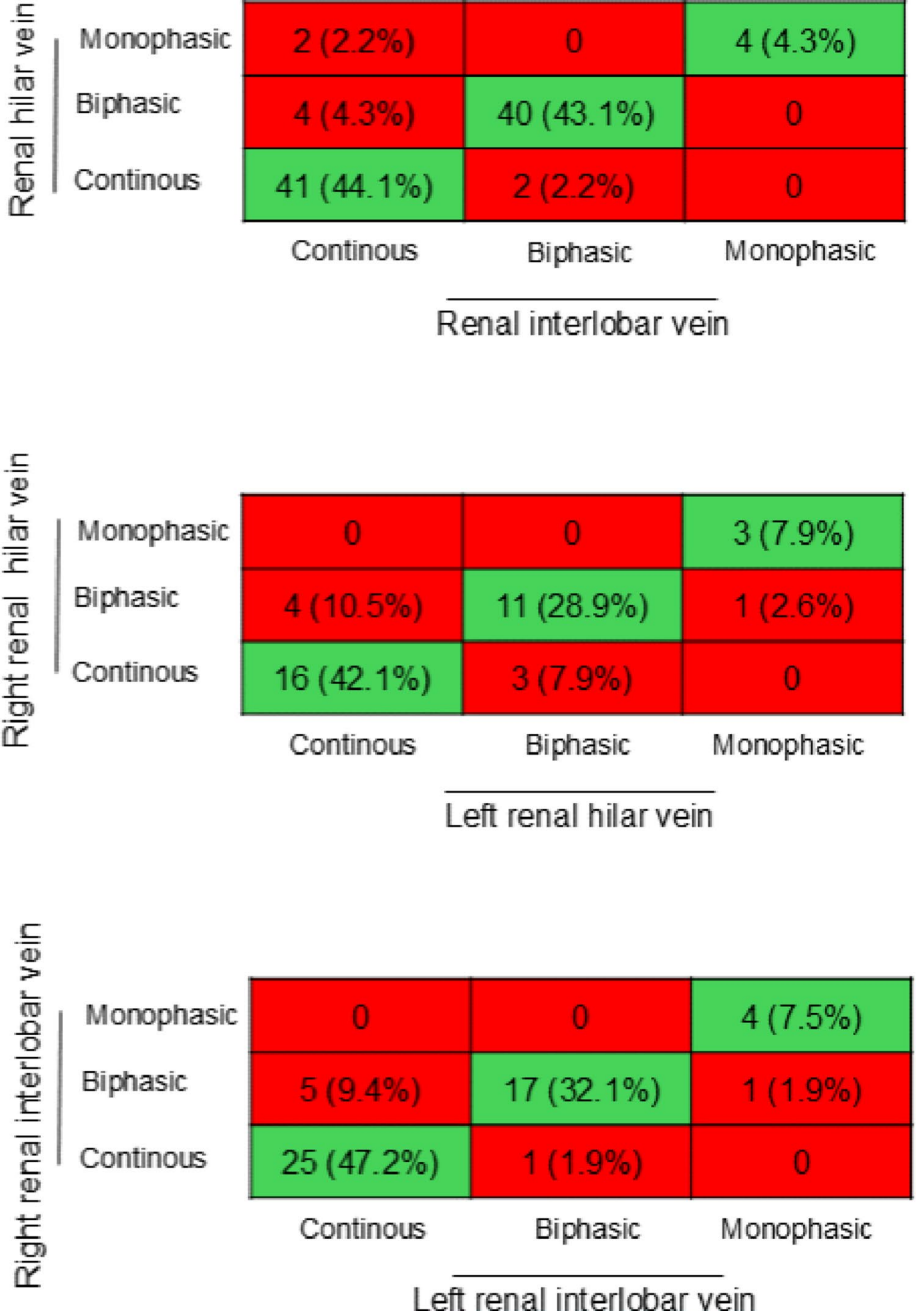
Comparison of renal venous flow patterns obtained in different anatomical locations. Green boxes represent agreement and red boxes represent disagreement

Renal venous hilar and interlobar flow patterns using measurements in kidneys with the best image quality were compared from 93 patients. The concordance rate was 91%, with a Cohen’s Kappa of 0.84, indicating strong agreement. In the comparison of hilar flow patterns between the kidneys in 38 patients, the concordance rate was 79% and Cohen’s Kappa was 0.63, indicating moderate agreement. Similarly, interlobar flow patterns compared between the right and left kidneys in 53 patients showed an 87% concordance rate and a Cohen’s Kappa of 0.77, also indicating moderate agreement. The specific distribution of agreements and disagreements is visualized in Fig. 1.

### Renal venous stasis index

**Fig. 2:**
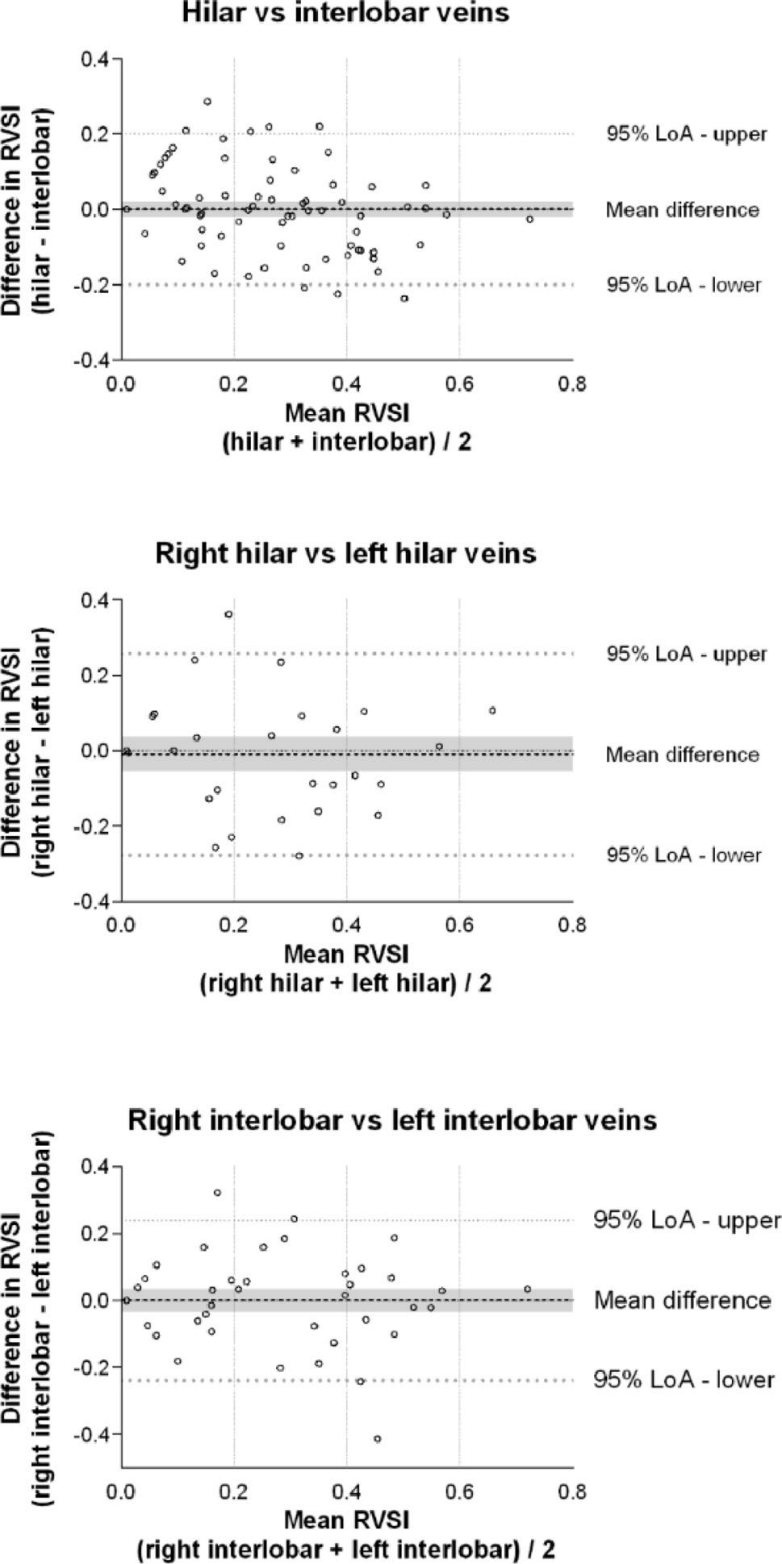
Comparison of renal venous stasis index measurements. The differences in each set of measurements are plotted on the y-axis. The mean values of each set of measurements are plotted on the x-axis. The grey area represents the 95% confidence interval of the mean difference

Renal venous stasis index values measured in the hilar and interlobar veins were compared using data from 94 patients. The mean bias was 0.00 (95% CI: − 0.02 to 0.02), with 95% LoA ranging from − 0.20 to 0.20 (Fig. 2). The bias constituted 0.2% of the overall mean renal venous stasis index, and the percentage error was 96%.

A comparison of renal venous stasis index between the right and left hilar veins was performed in 36 patients. The analysis yielded a mean bias of − 0.01 (95% CI: − 0.06 to 0.04), with 95% LoA between − 0.28 and 0.26 (Fig. 2). This bias represented 4.8% of the mean renal venous stasis index, while the percentage error was 133%.

In 52 patients the renal venous stasis index measurements from the interlobar veins of the right and left kidneys were compared. The mean bias was 0.00 (95% CI: − 0.03 to 0.03), with 95% LoA from − 0.24 to 0.24 (Fig. 2). This corresponded to a bias of 1.8% and a percentage error of 113%.

### Resistive index

**Fig. 3:**
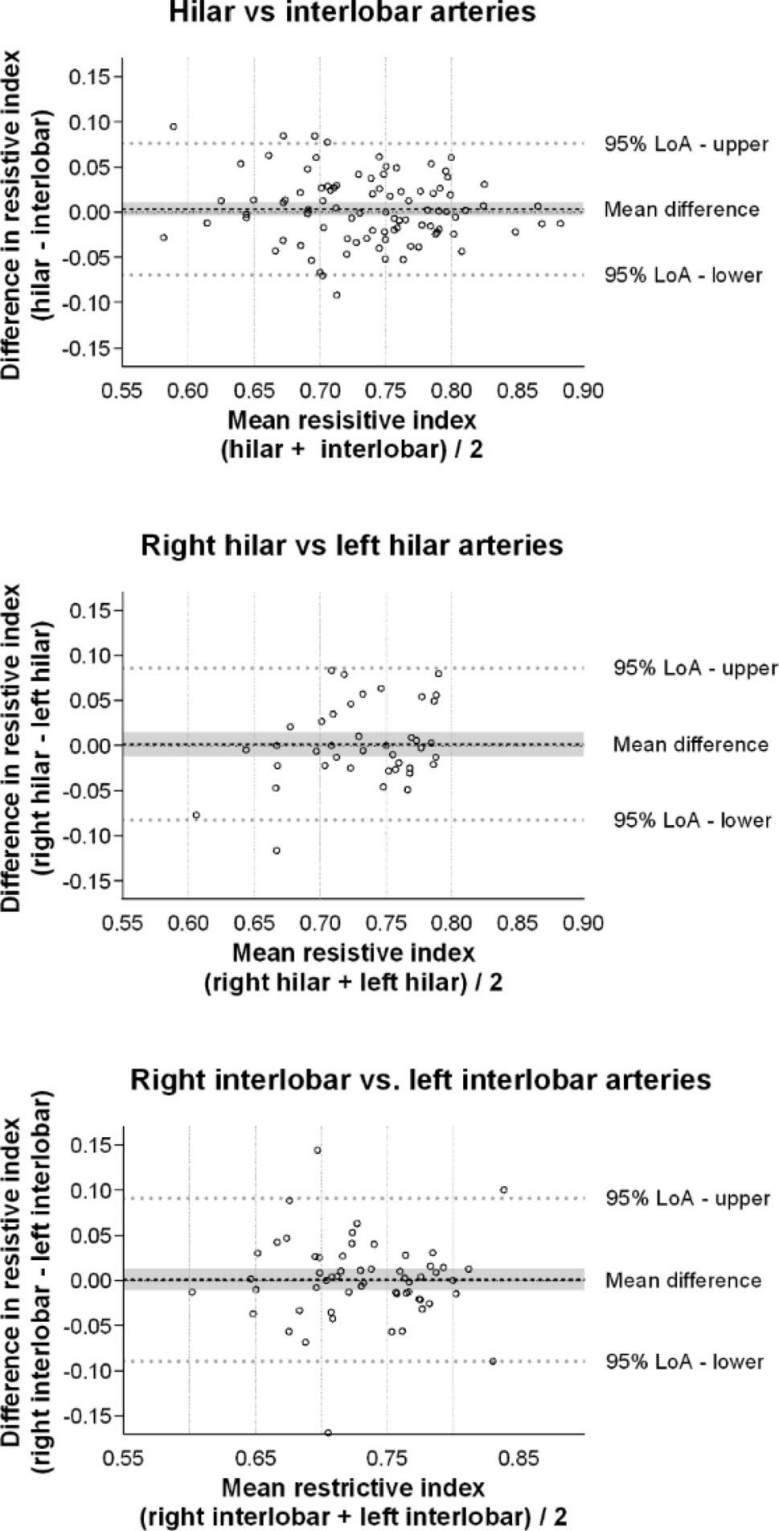
Comparisons of arterial resistive index measurements. The differences in each set of measurements are plotted on the y-axis. The mean values of each set of measurements are plotted on the x-axis. The grey area represents the 95% confidence interval of the mean difference

Resistive index measurements from 92 patients were used to compare hilar and interlobar arteries in the kidney with the best image quality. The mean bias was 0.00 (95% CI: − 0.00 to 0.01), with 95% LoA from − 0.07 to 0.08 (Fig. 3). The bias was 0.4% of the mean resistive index, while percentage error was 9.9%.

For comparisons between the hilar arteries of the right and left kidneys in 40 patients, the mean bias was 0.00 (95% CI: − 0.01 to 0.02), and the 95% LoA ranged from − 0.08 to 0.09 (Fig. 3). The percentage bias was 0.2%, and the percentage error was 11.5%.

In the comparison of interlobar arteries between kidneys in 57 patients, the mean bias was 0.00 (95% CI: − 0.01 to 0.01), with 95% LoA from − 0.09 to 0.09 (Fig. 3). This corresponded to a bias of 0.1% and a percentage error of 12.3%.

## Discussion

This study is the first to compare renal Doppler ultrasound measurements of arterial and venous organ flow in patients recovering from open-heart surgery. For the renal venous flow patterns, we observed strong agreement and a concordance rate higher than the predefined threshold of 90% when comparing hilar and interlobar veins, but only moderate agreement and a concordance rate below 90% was found when comparing measurements from the right and left kidneys. Continuous and biphasic flow patterns were most frequently misclassified when comparing the right and left kidney. In regard to the renal venous stasis index, we found minimal biases, below the predefined threshold of 10%, for hilar and interlobar vessels as well when comparing the right and left kidneys, but the limits of agreement were wide and the percentage error very high. For the resistive index, the bias was minimal, the limits of agreement were narrow, and the percentage error was low both when comparing hilar and interlobar arteries and right or left kidney.

We are not aware of any previous studies comparing renal venous Doppler measurements obtained in different anatomical locations.

For the arterial resistive index, the low biases accompanied by narrow limits of agreement for all comparisons indicate not only the absence of systematic errors and high accuracy but also high precision. These findings are in accordance with previous studies performed on healthy volunteers. Knapp et al. found that absolute renal arterial flow velocities decreased as measurements were performed more and more distally in the renal arterial tree. However, the decreases were almost identical for both systolic- and end-diastolic Doppler flows resulting in a minimal difference in resistive index [15]. Both Abe et al. and Baumgartner et al. found lower absolute arterial flow velocities in the left kidney as compared to the right kidney but found no difference in the arterial resistive index [12]. Baumgartner et al. also showed that this difference in absolute flow velocities from the right kidney to the left kidney could be attributed to the angle of insonation between the ultrasound probe and the target vessel.

Our study also revealed that reliable Doppler images from the left kidney could be obtained from a markedly lower percentage of patients as compared to the right kidney. This discrepancy may be attributed to the more dorsal anatomical position of the left kidney, combined with the superior acoustic window of the right kidney through the liver.

The high variability in the magnitude of differences in each set of renal venous stasis index measurements is noticeable. All analyses were based on images that were deemed of acceptable diagnostic quality; however, some variability in image quality was present, which may have resulted in random variation in the measurements. Averaging multiple Doppler samples and measurements at each site may counter this effect. Unfortunately, no validated method currently exists to quantify image quality or its variability in this specific context.

### Clinical implications

Our results indicate that when determining renal venous flow pattern, comparable results can be expected from hilar and interlobar veins. When comparing renal venous flow patterns sampled in the right and left kidney, the agreement was moderate mostly due to different classifications of continuous- and biphasic flow (Fig. 2). However, if using the VExUS ultrasound score for assessing venous congestion, a change from renal continuous flow to biphasic flow does not alter the final venous congestion score [3], as the score is mainly dependent on the number of severely abnormal organ flow patterns. When comparing renal venous stasis index low bias can be expected between both hilar and interlobar measurements and measurements in the right and left kidney. But there is high variability in magnitude of difference in each set of measurements, as the percentage errors were high and often exceeded 100%. This percentage error is clinically unacceptable, and sample locations are therefore not interchangeable for renal venous stasis index. Further our results show that when determining arterial resistive index, it is of limited importance whether sampling is performed in hilar- or interlobar arteries in either kidney. Finally, the right kidney generally provides more reliable Doppler curves. It therefore seems time-sparing to start with the right kidney and, if sufficient imaging is obtained, it does not provide additional information to scan the left kidney as well.

### Limitations

Each patient was scanned by a sonographer, who was not blinded to the ultrasound results obtained from other renal vessels on the same patient. This could theoretically have affected results in a direction of less bias and variation. However, image analysis was performed off-line by a reviewer who did not know which patient images were sampled from. Further, our hypothesis of differences in bias < 10%, percentage error < 30% or, for categorical data, concordance rates > 90% were based on clinical judgment. There is no objective standard defining adequate agreement between the ultrasound methods compared.

## Conclusion

In a population of postoperative open-heart cardiac surgery patients, comparable results can be expected when sampling renal venous flow pattern from hilar and interlobar veins. Overall low bias but also low precision can be expected when sampling RVSI in hilar and interlobar veins on either side. In regard to RI, low bias and high precision can be expected when sampling from hilar and interlobar arteries on either side. Overall, the image quality was superior in the right kidney.

## Supplementary Information

Below is the link to the electronic supplementary material.

Supplementary Material 1

## Data Availability

All data supporting the findings of this study are available within the paper and its Supplementary Information.

## References

[1] Darmon M et al. Diagnostic accuracy of doppler renal resistive index for reversibility of acute kidney injury in critically ill patients. Intensive Care Med, 2011. 37(1). 10.1007/s00134-010-2050-y 20862450

[2] Hermansen JL et al. Perioperative doppler measurements of renal perfusion are associated with acute kidney injury in patients undergoing cardiac surgery. Sci Rep, 2021. 11(1). 10.1038/s41598-021-99141-y PMC8492663 34611205

[3] Beaubien-Souligny W et al. Quantifying systemic congestion with Point-Of-Care ultrasound: development of the venous excess ultrasound grading system. Ultrasound J, 2020. 12(1). 10.1186/s13089-020-00163-w PMC7142196 32270297

[4] Beaubien-Souligny W et al. *Alterations in portal vein flow and intrarenal venous flow are associated with acute kidney injury after cardiac surgery: A prospective observational cohort study.* Journal of the American Heart Association, 2018. 7(19). 10.1161/JAHA.118.009961 PMC6404886 30371304

[5] Deschamps J et al. *Venous Doppler to Assess Congestion: A Comprehensive Review of Current Evidence and Nomenclature*, in *Ultrasound in Medicine and Biology*. 2023. 10.1016/j.ultrasmedbio.2022.07.011 36207224

[6] Gelman S. *Venous function and central venous pressure: a physiologic story.* (1528 – 1175 (Electronic)). 10.1097/ALN.0b013e3181672607 18362606

[7] Utrilla-Alvarez JD, et al. Assessing the venous system: correlation of mean systemic filling pressure with the venous excess ultrasound grading system in cardiac surgery. Echocardiography. 2023;40(11):1216–26. 37742087 10.1111/echo.15697

[8] Husain-Syed F et al. Doppler-Derived renal venous stasis index in the prognosis of right heart failure. J Am Heart Association, 2019. 8(21). 10.1161/JAHA.119.013584 PMC6898799 31630601

[9] Tublin ME, Bude RO, Platt JF. Review. The resistive index in renal doppler sonography: where do we stand? AJR Am J Roentgenol. 2003;180(4):885–92. 12646425 10.2214/ajr.180.4.1800885

[10] Arévalo Pérez J, et al. Angio CT assessment of anatomical variants in renal vasculature: its importance in the living donor. Insights Imaging. 2013;4(2):199–211. 23355302 10.1007/s13244-012-0217-5 PMC3609954

[11] Trpkov C, Grant ADM, Fine NM. Intrarenal doppler ultrasound renal venous stasis index correlates with acute cardiorenal syndrome in patients with acute decompensated heart failure. CJC Open. 2021;3(12):1444–52. 34993456 10.1016/j.cjco.2021.07.010 PMC8712550

[12] Abe M et al. Influence of renal function and demographic data on intrarenal doppler ultrasonography. PLoS ONE, 2019. 14(8). 10.1371/journal.pone.0221244 PMC6711528 31454365

[13] Renberg M et al. Feasibility of renal resistive index measurements performed by an intermediate and novice sonographer in a volunteer population. Ultrasound J, 2020. 12(1). 10.1186/s13089-020-00175-6 PMC7237552 32430724

[14] McHugh ML. Interrater reliability: the kappa statistic. Biochemia Med, 2012. 22(3). PMC3900052 23092060

[15] Knapp R, et al. Variability of Doppler parameters in the healthy kidney: an anatomic- physiologic correlation. J Ultrasound Med. 1995. 10.7863/jum.1995.14.6.427. 7658509 10.7863/jum.1995.14.6.427

